# Targeting gangliosides to treat Alzheimer’s and Parkinson’s diseases: A disruptive approach with the first-in-class peptide AmyP53

**DOI:** 10.4103/NRR.NRR-D-25-00076

**Published:** 2025-06-19

**Authors:** Jacques Fantini, Nouara Yahi

**Affiliations:** Faculty of Medicine, INSERM UA 16, Aix-Marseille University, Marseille, France

Neurodegenerative diseases are a growing burden on healthcare systems. Patients with Alzheimer’s or Parkinson’s diseases (AD or PD) are desperately waiting for innovative solutions that are slow to come, despite several decades of research worldwide. In 2021 and again in 2023, two monoclonal antibodies, aducanumab and lecanemab, have been approved by the U.S. Food and Drug Administration, and a third, donanemab, is currently under review. However, these treatments have very limited efficacy on cognitive functions and are accompanied by major side effects: amyloid-related imaging abnormalities, microhemorrhages, and accelerated brain volume loss (Høilund-Carlsen et al., 2024). The paradigm underlying these treatments is the amyloid cascade leading to the accumulation of amyloid plaques in the brain of patients (Fantini et al., 2020). Even if this strategy remains favored by most pharmaceutical companies, it suffers from major contradictions that could eventually lead to its abandonment. If the involvement of the Alzheimer’s amyloid-β (Aβ) protein remains undeniable, the debate has been open for several years to unequivocally identify the neurotoxic form of this protein. In fact, the presence of amyloid plaques is not systematically associated with AD, and we know of at least one deletion in the Aβ protein (Osaka mutation noted E22del or E22Δ) associated with the disease but without amyloid plaque (Fantini et al., 2020).

**Figure 1:**
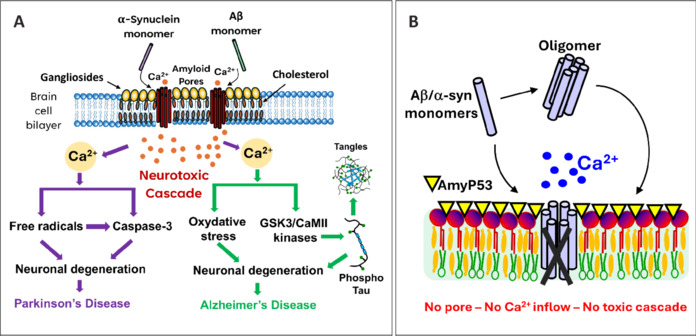
Common root cause of Alzheimer’s and Parkinson’s diseases. (A) The neurotoxic cascade is triggered by the massive entry of Ca^2+^ ions into brain cells, after the formation of amyloid-β (Aβ) (Alzheimer’s disease) or α-synuclein (α-syn) (Parkinson’s disease) oligomers. Neuronal degeneration is caused by the activation of specific enzymes such as caspase-3, glycogen synthase kinase-3 (GSK3), and Ca^2+^/calmodulin-dependent protein kinase II (CaMII). Tau hyperphosphorylation also contributes to neuronal degeneration and tangle formation. The initial event of the cascade is the interaction of a monomeric amyloid protein with raft gangliosides. Then the protein inserts into the membrane, recruits other monomers, and self-assembles into an oligomeric amyloid pore under the control of cholesterol. (B) The unique molecular mechanism of AmyP53. The AmyP53 peptide interacts with gangliosides and prevents any binding of Aβ or α-syn monomers and oligomers to the brain cell membrane, thereby blocking the neurotoxic cascade at its earliest step.

The guilt of amyloid plaques is therefore not proven, and this should encourage us to question the paradigm. Indeed, over an average lifespan, there are more people with amyloid plaques but without dementia than people with amyloid plaques and dementia (Jack et al., 2019). For over thirty years, a bundle of converging arguments has made it possible to identify small oligomers of Aβ protein as the neurotoxic form actually causing the symptoms (Fantini et al., 2020). Among the different types of oligomers, those forming pores selectively permeable to calcium ions in the membrane of brain cells are considered the most toxic species of Aβ protein. Gangliosides and cholesterol in lipid raft microdomains are key players in the generation of neurotoxic oligomers formed by Aβ (AD) (Fantini et al., 2020) and α-synuclein (PD) in neural membranes (Yahi et al., 2022). The massive entry of Ca^2+^ ions into the cells then triggers a cascade of events including all the hallmarks of AD and PD, especially hyperphosphorylation of Tau protein, oxidative stress, neuronal degeneration, and then neuronal loss (**Figure 1A**).

Blocking this neurotoxic cascade therefore appears to be a strategy of choice for treating both AD and PD (**Figure 1B**; Fantini et al., 2020; Yahi et al., 2022). It is with this objective that our team undertook, about twenty years ago, to elucidate the molecular mechanisms controlling the formation of oligomeric amyloid pores. This is how we have highlighted the role of lipid rafts and more precisely that of gangliosides and membrane cholesterol (Fantini et al., 2020). Briefly, the amyloid protein is first attracted by the raft, binds to a ganglioside, and then inserts into the membrane where it can interact with cholesterol which catalyzes the oligomerization process. Blocking the phenomenon at its earliest stage is possible if we manage to prevent any interaction between amyloid proteins and raft gangliosides (**Figure 1B**).

The identification of the interaction domain of Aβ with raft gangliosides was a long-term work, because Aβ belongs to the category of intrinsically disordered proteins, a class of proteins that do not adopt a stable structure in solution and control multiple cellular mechanisms such as cell proliferation and differentiation (Uversky et al., 2008). Additionally, the Aβ protein does not interact with a single ganglioside, but with several, which complicated the task and required the development of experimental methods taking this particularity into account. Furthermore, raft gangliosides are mobile elements that cannot be reduced to an average conformation that could be used as a reliable starting condition in molecular modeling studies. It was therefore not a question of identifying complementary structures that, by definition, do not preexist either in the Aβ protein or in raft gangliosides. Paradoxically, we solved the problem by introducing another interactome in the study, i.e., the α-synuclein/ganglioside complex. More precisely, our strategy was to compare the ganglioside recognition properties of Aβ and α-synuclein rather than limiting our study to a single protein/ganglioside system. These two proteins interact with raft gangliosides and form neurotoxic oligomers in neural cell membranes under the control of cholesterol. We first identified the ganglioside preferentially recognized by each of these proteins, which appeared to be GM1 for Aβ and GM3 for α-synuclein (Fantini et al., 2025). Then we analyzed these protein-ganglioside interactions with synthetic peptides derived from both proteins, which led us to identify a disordered 12-amino acid domain capable of structuring itself into a functional ganglioside-binding domain (Fantini et al., 2025). The last step consisted of identifying, by alanine scanning, the amino acids controlling the specific interaction with the preferential ganglioside of each of the two proteins. Finally, we combined all this information into a chimeric peptide capable of recognizing GM1 and GM3 gangliosides with the same affinity. We thus created in a completely rational manner the first anti-ganglioside molecule, the therapeutic peptide AmyP53, whose design is based on the decryption of a biological code (Fantini et al., 2025). This code controls the formation of neurotoxic oligomers of amyloid proteins associated with AD and PD. The peptide AmyP53 is the first representative of a new category of therapeutic molecules, adaptive peptides (Fantini et al., 2025). These adaptive peptides are mini-intrinsically disordered proteins that do not have a particular structure before interacting with gangliosides (Fantini et al., 2025). In this sense, they mimic the conformational flexibility properties of the disordered proteins from which they are derived (Uversky et al., 2008). AmyP53 effectively blocks any interaction of Aβ and α-synuclein proteins with raft gangliosides and consequently the entire neurotoxicity cascade that results from it: formation of oligomeric amyloid pores, entry of Ca^2+^ ions into brain cells, hyperphosphorylation of the Tau protein, oxidative stress, neuronal degeneration, and death (**Figure 1B**). It can be administered to nerve cell cultures as a synthetic peptide or by gene therapy with a retroviral vector designed to synthesize and secrete the peptide into the culture medium (El-Battari et al., 2021). These cells are then completely protected from the neurotoxic effects of Aβ and α-synuclein proteins. AmyP53 also preserves the neuronal networks of the hippocampus from the deleterious effects of Aβ oligomers (Fantini et al., 2025). Lacking a proteolytic cleavage site, AmyP53 is remarkably stable (Di Scala et al., 2022). When administered intravenously, it rapidly reaches the brain due to its blood-brain barrier crossing properties. Yet the intravenous route is not the one we are considering for future clinical trials in humans. Interestingly, the amount of peptide reaching the brain is even greater when the peptide is administered intranasally (Di Scala et al., 2022). It is therefore with a nasal spray dedicated to the nose-to-brain route that we are going to administer the AmyP53 peptide in clinical trials. Among other advantages, the high-water solubility of AmyP53 (up to 200 mg/mL) is compatible with a stable formulation in a nasal spray device.

**Figure 2:**
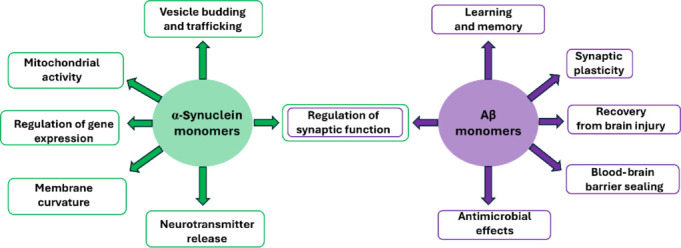
Main physiological functions of α-synuclein and amyloid-β (Aβ) monomers.

AmyP53 is innovative in more than one way. It comes from the decryption of a biological code that controls the neurotoxicity of amyloid proteins responsible for AD and PD. This code determines the mode of association of these proteins with the gangliosides of the plasma membrane of brain cells. This interaction is the very first step of the neurotoxic cascade initiated by the massive entry of Ca^2+^ ions into the cells. AmyP53 blocks this process by preventing the association of Aβ and α-synuclein proteins with gangliosides. It also blocks the insertion of preformed soluble oligomers which is also a ganglioside-dependent process (Fantini et al., 2020; **Figure 1B**). The molecular mechanism that determines the therapeutic effect of the AmyP53 peptide is totally innovative, since it is based on the conformational flexibility properties inherited from the parental amyloid proteins that served as a model for its design. In this regard, AmyP53 is the first representative of adaptive peptides. As discussed above, adaptive peptides do not have a precise 3D structure in solution, but they are progressively structured during their interaction with the ligand, in this case, the gangliosides of lipid rafts (Fantini et al., 2025). Targeting gangliosides to treat neurodegenerative diseases is a disruptive approach that challenges the old paradigm that considers amyloid plaques and Lewy bodies as pathogenic aggregates (Fantini et al., 2020; Yahi et al., 2022). Instead, AmyP53 tackles the root cause of AD and PD, i.e., neurotoxic oligomers. Most importantly, this strategy preserves the physiological functions of amyloid proteins, especially those involved in memory control (Aβ) and the synaptic vesicle cycle (α-synuclein; **Figure 2**). Those amyloid proteins can exert not just one but several physiological functions that are directly linked to their disordered nature. These intrinsically disordered proteins lack a stable 3D structure, and they can adopt several distinct biologically active conformations allowing them to exert a broad range of functions. Under these conditions, it is clearly hazardous to act on their homeostasis or target them with immunotherapies. As a matter of fact, this is why strategies aimed at decreasing Aβ levels with secretase inhibitors are so toxic and unsuccessful (“worse than placebo”; Fantini et al., 2020).

By combining the ganglioside-binding properties of two proteins (α-synuclein and Aβ), AmyP53 can be considered a therapeutic combination formulated in a single molecule, which is also a major innovation (Fantini et al., 2020; Yahi et al., 2022). This property may be decisive for patients with Aβ/α-synuclein co-pathologies. Moreover, there are undeniable advantages for therapeutic peptides: target specificity, low dose required to obtain a therapeutic effect, low toxicity, metabolism into natural amino acids recycled in protein biosynthesis, and ease of large-scale manufacturing with low production costs. The intranasal route of administration allows direct access to the brain while bypassing the bloodstream, which helps to limit systemic adverse effects.

How can AmyP53 be positioned in the drug pipeline for patients with AD or PD? Clinical trials will help to clarify which patients are most likely to benefit from this new type of therapy. If we compare these diseases to a truck speeding down a road, it seems reasonable to slow down the truck and stop the engine. In this way, the primary cause of the malfunction will cease. Will the damage already present be reversible? The unique mechanism of AmyP53 allows for a combination therapy with any neuroprotective strategy, including improving brain plasticity by dedicated drugs, stem cells, or neuro-implants. The demonstrated reversibility of the toxic effects of Aβ protein oligomers on cultured neurons also suggests the intriguing notion that recovering certain brain functions through brain plasticity mechanisms should be possible (Tanokashira et al., 2017). AmyP53 could also be used as a preventative strategy for patients with mutations associated with AD and PD diseases in the genes encoding Aβ and α-synuclein (Di Scala et al., 2016). In marked contrast with current anti-plaque immunotherapies, based on monoclonal antibodies that recognize several forms of Aβ (unfortunately including monomers), AmyP53 does not target the protein, but the pool of gangliosides that is specifically associated with the initiation of the neurotoxic cascade in pathological conditions. Thus, with our strategy, the physiological role of the monomeric forms of the amyloid proteins is not affected. Indeed, the preclinical data have demonstrated the exceptional tolerability profile of AmyP53. Nevertheless, the safety of targeting gangliosides with AmyP53 warrants a careful examination. Firstly, it is known that gangliosides play a crucial role in modulating axon-myelin interactions. Thus, it is important to consider whether AmyP53 might disrupt these essential processes. Secondly, gangliosides control the function of several membrane receptors by modulating their conformation through chaperone-like effects in lipid raft microdomains. As discussed by Krengel and Bousquet (2014), this effect implies that the head groups of these gangliosides are not accessible in *trans* to extracellular ligands, precisely because they are masked by the membrane receptors with which they are physically associated. In contrast, AmyP53 targets a distinct pool of gangliosides that are not associated with membrane proteins and are thus accessible to extracellular ligands. This excludes any functional interference with regulatory gangliosides associated with myelin or membrane receptors (Fantini, 2023).

One might also ask whether AmyP53 could interfere with the normal cellular functions of amyloid proteins. This is unlikely because AmyP53 is very well tolerated in animals, with a NOAEL > 80 mg/kg body weight (intravenous administration) and > 5 mg/kg body weight (intranasal administration). These values actually correspond to the maximum doses injected in maximum tolerated dose and dose ranging finding studies (El-Battari et al., 2021). Moreover, intranasal administration of AmyP53 (5 mg/kg body weight in rats) did not induce any neurologic adverse effects on behavior and physiological function by the Primary Observation Test (Irwin Test).

Based on these preclinical data in rodent and nonrodent animal species, AmyP53 is not expected to induce any toxic side effects. Preclinical studies also showed that repeated intranasal administration of AmyP53 in rabbits did not induce the production of anti-drug antibodies that could limit the clinical use of the peptide. This is consistent with the fact that AmyP53 is derived from a fragment of human α-synuclein which has a significant overlap of sequence with rabbit alpha-synuclein (75% of homology) and 83% homology with rat and mouse alpha-synuclein. Thus, we do not expect any immune response induced by AmyP53 in clinical trials.

In summary, AmyP53 is a disease modifying drug that opens the route for the prevention and/or treatment of AD and PD in clinical trials, bringing a new hope for patients, families, and caregivers.

*JF and NY are co-inventors of the AmyP53 peptide (patent Application EP15709163.8A), currently under development for the treatment of Alzheimer’s and Parkinson’s diseases by the AmyPore Company. No conflicts of interest exist between AmyPore Company and publication of this paper.*

**Additional file:** *Open peer review report 1.*

OPEN PEER REVIEW REPORT 1
