# Supplementary material for: Targeting gangliosides to treat Alzheimer’s and Parkinson’s diseases: A disruptive approach with the first-in-class peptide AmyP53
Source: Neural Regen Res. 2025 Jun 19;21(6):2339–40. doi: 10.4103/NRR.NRR-D-25-00076 (PMC13211832; doi:10.4103/NRR.NRR-D-25-00076)
Supplement: Supplementary file 1 [file NRR-21-2339_Suppl1.pdf]

OPEN PEER REVIEW REPORT 1

Name of journal: Neural Regeneration Research
Manuscript NO: NRR-D-25-00076
Title: Targeting gangliosides to treat Alzheimer's and Parkinson's diseases: a disruptive approach with the first-in-class peptide AmyP53
Reviewer's Name: Nisi Jiang
Reviewer's country: USA

COMMENTS TO AUTHORS

The authors present a compelling and intriguing perspective on targeting gangliosides as a potential therapeutic strategy for Alzheimer's and Parkinson's diseases.

One suggestion for improvement is to address the potential side effects of targeting gangliosides. Specifically, if AmyP53 blocks the binding of Aβ and α-synuclein, could this interfere with the normal intracellular functions of these proteins, potentially leading to unintended consequences? Additionally, as gangliosides play a crucial role in modulating axon-myelin interactions, it would be important to consider whether AmyP53 might disrupt these essential processes. Including a paragraph discussing these potential side effects would enhance the manuscript's comprehensiveness and strengthen its overall impact.

Lastly, a minor point: there appears to be repetitive text in Page 4, lines 44-48, and Page 5, lines 1-5. Removing or consolidating this text would improve the manuscript's readability.
